# Reactive hyperperfusion after short gastric artery division: a real-time indocyanine green fluorescence-guided approach to laparoscopic fundoplication – a case report

**DOI:** 10.1097/RC9.0000000000000819

**Published:** 2026-08-06

**Authors:** Simeng Li, Lee S Kyang, Nurojan Vivekanandamoorthy, David Goltsman, Aldenb Lorenzo

**Affiliations:** a Department of Surgery, Bankstown Hospital, Sydney, Australia; b School of Medicine, Sydney Medical School, University of Sydney, Sydney, NSW, Australia; c School of Medicine, University of New South Wales, Sydney, NSW, Australia

**Keywords:** complication, dysphagia, indocyanine green, laparoscopic fundoplication

## Abstract

**Introduction:**

Fundoplication is an effective surgical treatment for gastroesophageal reflux disease, but postoperative dysphagia remains a common complication; intraoperative use of indocyanine green (ICG) fluorescence imaging allows real-time assessment of perfusion and guides surgical decision-making.

**Case presentation:**

A 38-year-old male underwent laparoscopic hiatus hernia repair and fundoplication for persistent reflux symptoms despite maximal medical therapy. ICG was administered intravenously at three time points: after port placement, following division of the short gastric arteries, and during wrap formation. After division of the short gastric arteries, a bright orange fluorescence was observed in the stomach fundus, indicating reactive hyperemia. This could suggest an autoregulatory response to maintain perfusion. The patient remained symptomatically well without dysphagia or reflux at 6-month follow-up.

**Discussion:**

The real-time change in perfusion was then used to guide the formation of the fundoplication, and final imaging confirmed adequate vascularity of the wrap without compromising the anti-reflux effect of the surgery.

**Conclusion:**

ICG fluorescence imaging is a safe and effective adjunct to intraoperative assessment of gastric perfusion during laparoscopic fundoplication, serves as a valuable training tool for guiding wrap construction, and may prevent postoperative dysphagia.

## Introduction

Gastroesophageal reflux disease (GERD) is one of the most common gastrointestinal disorders, with a prevalence of up to 30%^[^1^]^. Medical therapy is the first-line treatment. Approximately 30% of patients taking proton pump inhibitors do not achieve adequate symptom control^[^2^]^. Fundoplication is currently the “gold-standard” for treating GERD in patients who do not respond completely to medications^[^1^]^.

HIGHLIGHTS

- ICG fluorescence provides real-time intraoperative assessment of gastric and wrap perfusion during laparoscopic fundoplication.
- Reactive hyperemia was observed after division of the short gastric artery.
- ICG may reduce the risk of post-fundoplication dysphagia by guiding the construction of the wrap.
- ICG can serve as a valuable training tool in fundoplication surgery.

Fundoplication is a surgical method in which the stomach’s fundus is wrapped around the intra-abdominal esophagus, thereby increasing the sphincter’s intra-abdominal length, narrowing the sphincter and accentuating the angle of His. Although it has been proven to be a very effective form of therapy, complications can occur. One of the most troublesome complications is dysphagia^[^3^]^. The causes of dysphagia include hiatal stenosis, anterior angulation of the gastroesophageal junction (GEJ) and an over-compensated fundoplication^[^3^]^.

Indocyanine green (ICG) fluorescence imaging, when visualized under near-infrared (NIR) light, offers a real-time, objective method to assess tissue vascularity. This article describes the use of ICG fluorescence at three distinct points during hiatus hernia repair and fundoplication to guide surgical decision-making and ensure adequate vascular supply to the wrap and esophagus.

## Case presentation

A 38-year-old male presented with persistent reflux symptoms despite maximal medical therapy. The symptoms were exacerbated by lying supine and at night. Gastroscopy revealed a 4-cm hiatus hernia with evidence of persistent reflux esophagitis. The patient was subsequently scheduled for laparoscopic repair of a hiatus hernia and a partial fundoplication. This case report has been prepared in line with the SCARE checklist^[^4^]^.

## Surgical technique

### ICG preparation

One vial of ICG contained 25 mg. This was diluted in 10 mL of normal saline, yielding a concentration of 2.5 mg/mL. At each stage of assessment, 2 mL of ICG was administered intravenously.

#### Baseline perfusion assessment

**Figure 1.**
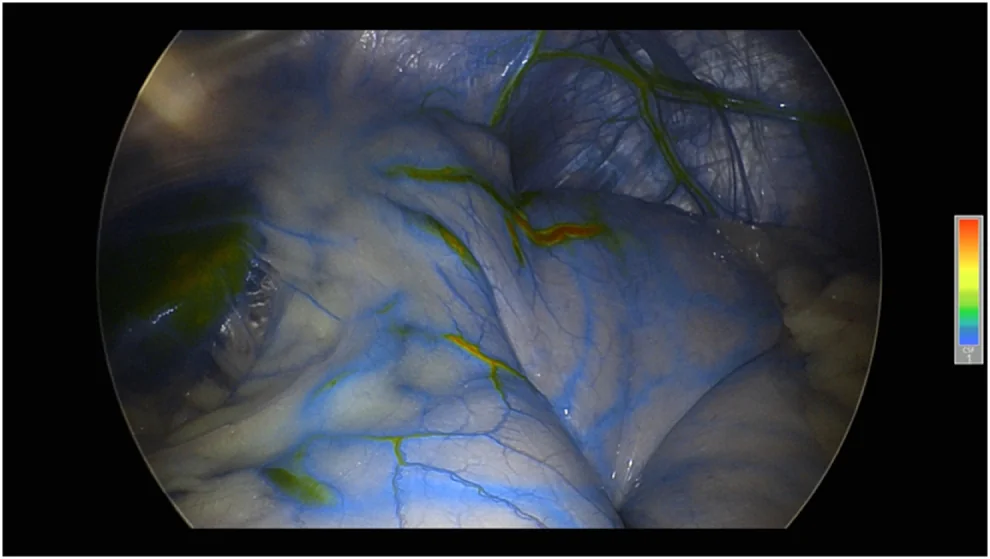
Baseline perfusion assessment. NIR imaging demonstrates a gradient of vascularity in the gastric fundus, with orange areas indicating well-perfused tissue and blue areas indicating relatively less vascularized regions.

In the operating room, the patient was positioned supine, with arms out, in a reverse Trendelenburg position, under general anesthesia. Optical entry with a 12-mm port was performed at Palmer’s point. The remaining ports were placed under direct visualization as follows: a 5-mm right subcostal, anterior axillary line; 12-mm left subcostal, anterior axillary line; and 5-mm left subcostal, mid-axillary line. A Nathanson retractor was placed to retract the liver. The first dose of 2 mL of ICG was administered intravenously. NIR imaging using the SPY CSF® (Stryker) mode was then activated. The resulting fluorescence demonstrated a gradient of perfusion and vascularity in the gastric fundus. Well-perfused areas appeared orange, while less-perfused regions appeared blue (Fig. 1). This ICG administration served as a baseline assessment of the perfusion of the gastric fundus and lower esophagus before mobilization.

#### Reactive hyperemia phenomenon after division of the short gastric artery

**Figure 2.**
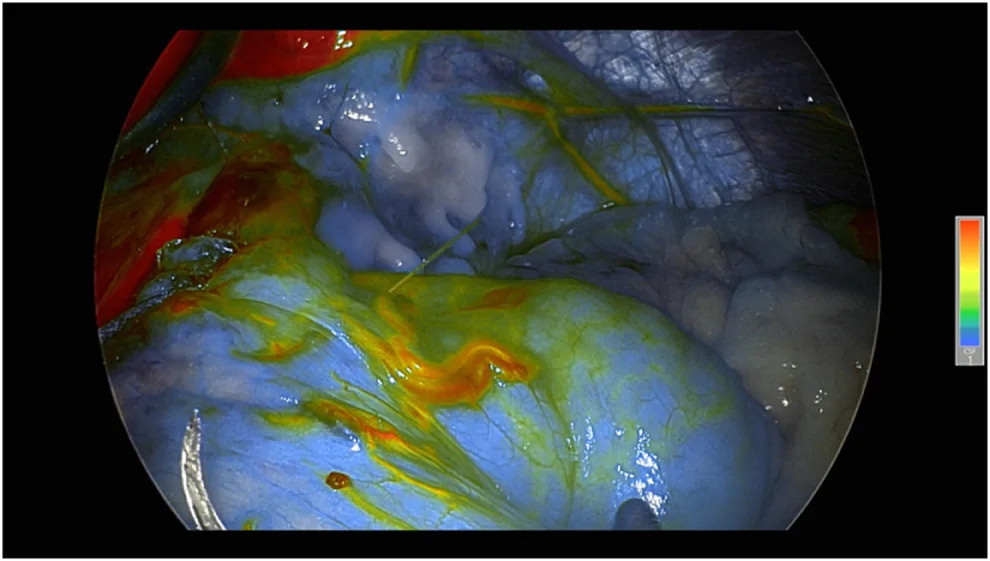
Reactive hyperperfusion after short gastric artery division. Following division of the short gastric vessels, a second 2-mL ICG injection revealed intense, bright orange fluorescence in the fundus. This represents a reactive hyperperfusion phenomenon, likely an autoregulatory response to transient hypoperfusion.

To mobilize the fundus, the pars flaccida was divided. Hiatal dissection was then performed, followed by division of the short gastric vessels. The point of division of the short gastric vessels was proximal to the line of the angle of His. A second dose of 2 mL of ICG was then administered intravenously. A bright orange fluorescence was observed in the stomach fundus, suggesting a phenomenon of reactive hyperemia (Fig. 2). Although the exact mechanism remains unknown, this is likely an autoregulatory response to transient hypoperfusion.

#### Wrap construction and final perfusion check

**Figure 3.**
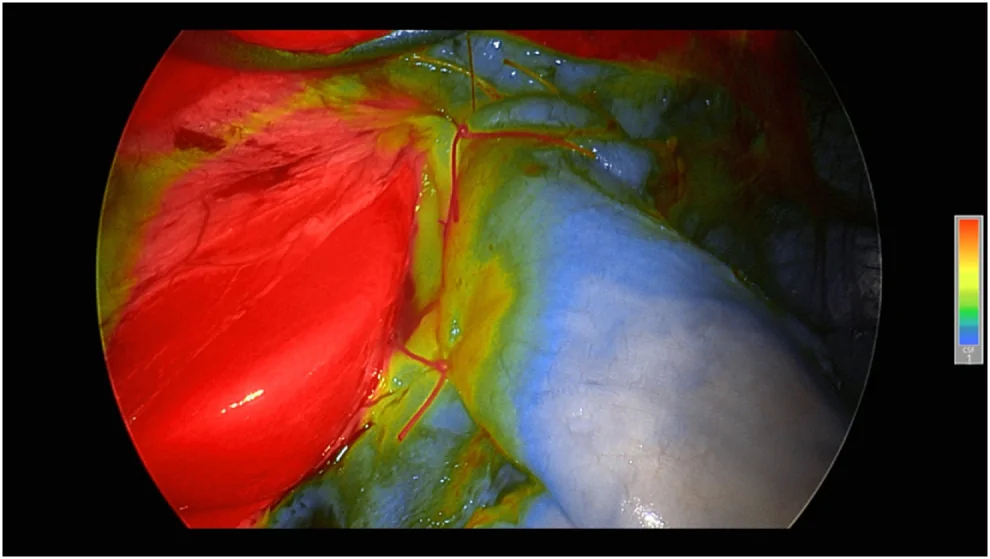
Wrap completion and perfusion verification. The final perfusion check was performed following completion of the fundoplication. After a third dose of ICG, NIR imaging demonstrated bright orange fluorescence along the wrap, confirming uniform perfusion and good vascularity. This intraoperative verification provided reassurance that the wrap had been constructed without ischemia.

Following fundal mobilization, the left hiatal pillar was identified, and a retro-esophageal space was created to accommodate an esophageal nylon tape for gentle retraction. Mediastinal dissection was completed to achieve a sufficient intra-abdominal esophagus length (3–4 cm). This length ensures adequate support for the wrap. A posterior cruroplasty was performed using non-absorbable Ethibond® (Ethicon, Johnson & Johnson) sutures. A butterfly-shaped Phasix® (CR Bard Inc., Murray Hill, NJ) mesh was placed and secured to the hiatal pillars at three points using 2-0 Prolene sutures. The angle of His was reconstructed using Ethibond® suture, after which a final dose of 2 mL of ICG was administered to assess fundal perfusion in real time during construction of a 180° anterior fundoplication wrap (Fig. 3). ICG fluorescence provided real-time visual assessment of fundal perfusion during construction of a 180° anterior fundoplication. The findings served as an adjunct to surgical judgement by confirming preserved vascularity throughout wrap formation and supporting construction of a tension-free fundoplication. The patient reported no symptoms suggestive of dysphagia or reflux at 6-month post-operative follow-up.

## Discussion

Postoperative dysphagia, especially for solid foods, is expected in patients for the first 2–6 weeks after fundoplication because of postsurgical edema or transient esophageal hypomotility^[^5,6^]^. Marked dysphagia for liquids is rare and should suggest anatomical dysfunction. These patients are often treated with dietary modification, and their dysphagia usually resolves within 2–3 months^[^7^]^. However, 3–44% of patients experience dysphagia that persists beyond 3 months^[^3^]^. Although the exact etiology remains unknown, it is thought to be related to a tight hiatus or slipped fundoplication^[^8,9^]^. Traditionally, during surgery, the tightness of the wrap is assessed by observing the ease of scope passage, the appearance and palpation of the wrap, and in some cases by using intraoperative manometry to measure GEJ pressures. However, these methods are either not readily available in most facilities or are reliant on subjective experience.

The use of ICG for the intraoperative evaluation of the GEJ has previously been documented in a few case reports, including cases involving sleeve gastrectomy and the laparoscopic resection of an esophageal duplication cyst^[^10,11^]^. To our knowledge, this is the first case to describe the application of ICG during hiatus hernia repair and fundoplication.

We propose the application of ICG fluorescence angiography in laparoscopic fundoplication as an adjunctive tool to enhance intraoperative assessment of tissue vascularity. In this case, fluorescence imaging provided real-time confirmation of preserved blood flow following fundal mobilization and short gastric vessel division. Although no change in operative strategy was required, the findings provided objective reassurance regarding tissue perfusion during wrap construction and supplemented the surgeon’s assessment when creating a tension-free fundoplication. The greatest clinical utility of ICG may be realized in situations where impaired perfusion is detected, and corrective intraoperative measures can be undertaken. This needs to be further proven and assessed in future case series. ICG use extends beyond clinical safety, as it could potentially improve surgical education. By providing an immediate visualization of vascular changes, the ICG technique allows trainees to better understand the physiological consequences of their operative maneuvers. This can serve as an invaluable adjunct in teaching wrap construction, reinforcing safe surgical principles.

Division of the short gastric arteries has remained controversial since the first fundoplication was performed a decade ago^[^12^]^. Proponents believe short gastric artery division allows the fundus to be fully mobilized and wrapped loosely around the esophagus, minimizing tension and the risk of postoperative dysphagia^[^12,13^]^. Although routine short gastric vessel division is not universally required during anterior fundoplication, complete fundal mobilization was performed in this case according to the operating surgeon’s standard technique to facilitate a tension-free wrap and the formation of the angle of His. However, opponents argue that the short gastric arteries contribute to the vascular supply of the fundus, and their division may increase the risk of gastric ischemia. In this case, we observed increased perfusion after vessel division. This demonstrates the stomach’s autoregulatory capacity to maintain blood flow via collateral circulation. Large-scale studies using standardized protocols are needed to confirm this physiological response and to correlate this phenomenon with post-operative dysphagia outcomes.

Several limitations should be acknowledged. First, ICG fluorescence imaging evaluates only visible surface perfusion and cannot directly assess transmural blood flow or the vascularity of obscured portions of the completed fundoplication, including the posterior aspect of the wrap and the esophagogastric interface. Second, satisfactory intraoperative fluorescence does not necessarily predict delayed ischemia, long-term vascular adequacy, or functional outcomes such as postoperative dysphagia. Third, interpretation of fluorescence intensity remains largely qualitative and operator dependent. Finally, corresponding high-resolution white-light images were unavailable for inclusion in this report. This limited detailed assessment of the final anatomical configuration. Further studies should incorporate both white-light and fluorescence imaging together with quantitative perfusion assessment and long-term clinical follow-up. This case highlights the novel use of ICG in laparoscopic repair of hiatal hernia and partial fundoplication. The real-time assessment of wrap construction guided by fluorescence imaging may help achieve optimal reflux control and avoid post-fundoplication dysphagia. While these findings are promising, there is a need for larger patient cohorts and prospective studies with standardized fluorescence quantification methods to validate the technique’s efficacy and safety. In addition, while ICG enables visualization of perfusion through color differentiation (orange versus blue), interpretation remains subjective. Future research is needed to develop quantitative platforms that can convert these color signals into objective, numerical perfusion values.

## Conclusion

This is a novel case of the application of intraoperative ICG fluorescence during laparoscopic fundoplication to help tailor the tension of the fundoplication wrap. This technology holds promise as a training tool, enabling surgeons in training to visualize perfusion dynamics while avoiding post-fundoplication dysphagia. Future studies are required to determine its ability to influence operative decision-making and improve long-term clinical outcomes.

## Data Availability

Not applicable.

## Artificial intelligence

The authors declare that no artificial intelligence was used in the writing.
